# *Bryum billardieri* Schwaegr. against EV71 infection: *in vitro* and *in vivo* antiviral effects, identification of molecular mechanisms and active monomers

**DOI:** 10.3389/fphar.2023.1164784

**Published:** 2023-08-15

**Authors:** Yun-Yu Wang, Qian Li, Xiu-Wei Han, Xin-Huan Wan, Li Zhang, Feng-Jv Niu, Yi-Zhou Xin, Chang-Zheng Zhou

**Affiliations:** ^1^ College of Pharmacy, Shandong University of Traditional Chinese Medicine, Ji’nan, China; ^2^ Shandong Qidu Pharmaceutical Co., Ji’nan, China; ^3^ Zhaoyuan Inspection and Testing Center, Yantai, China; ^4^ The Fifth Affiliated Hospital of Xinjiang Medical University, Urumqi, China; ^5^ Affiliated Hospital of Shandong University of Traditional Chinese Medicine, Ji’nan, China

**Keywords:** *Bryum billardieri* Schwaegr., EV71, toll-like receptors, cytokines, saponarin

## Abstract

Enterovirus 71 (EV71) commonly causes symptoms such as hand, foot, and mouth disease (HFMD) in infants and children and may lead to neurological disease and even death in severe cases. Appropriate vaccines for the prevention of HFMD are available in the clinic; however, they present different and serious adverse effects that cannot guarantee compliance and efficacy. The purpose of this study was to analyze the potential mechanism of *Bryum billardieri* Schwaegr. (BBS) against EV71 and analyze its potential active components. A previous *in vitro* antiviral assay was used to determine the best extraction method for the active site of BBS against EV71, and the results showed that the antiviral activity of BBS was more pronounced in the fraction that was extracted by aqueous extraction and alcoholic precipitation and then obtained by purification on a silica gel column (dichloromethane:methanol = 0:100). In addition, the therapeutic effects of BBS on EV71-infected mice were further investigated by *in vivo* pharmacological experiments. BBS reduced the lung index, viral titer, and degree of EV71-induced lung, brain, and skeletal muscle damage. The mechanism of anti-EV71 activity of BBS was also investigated by using ELISA and qRT-PCR, and it was found that BBS exerted its action mainly by regulating the expression of TLR3, TLR4, TNF-*α*, IL-2, and IFN-*γ* by modulating the activation of NF-*κ*B and JAK2/STAT1 signaling pathways. Finally, the chemical structures of the active monomers in BBS were determined by using UPLC-MS and NMR techniques. The study revealed that one of the monomers on which BBS exerts its antiviral activity is saponarin. In conclusion, the results of this study suggest that BBS is considered a natural anti-EV71 product with enormous potential, and saponarin would be its non-negligible active monomer.

## Graphical Abstract

**Figure:**
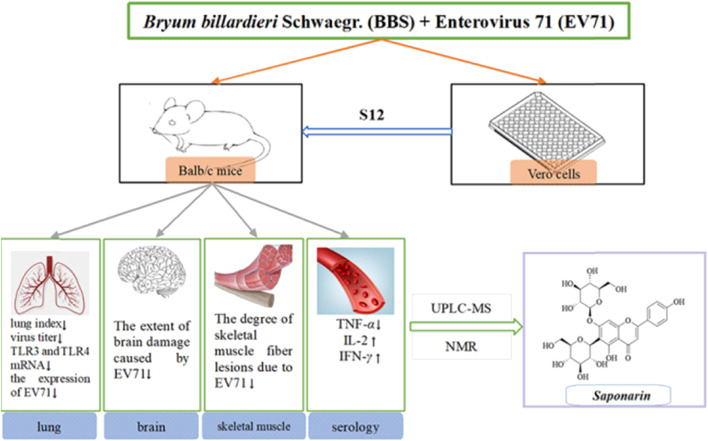

## 1 Introduction

EV71 is the main cause of acute hand, foot, and mouth disease (HFMD) infection in children, with the human brainstem being the leading target site of infection (Lin et al., 2003; Singh et al., 2006; Puenpa et al., 2019). In severe cases, it can cause complications such as meningitis, acute and delayed paralysis, pulmonary edema, and cardiopulmonary failure (Yin et al., 2019). The human enterovirus genus has four main serotypes (A, B, C, and D), among which EV71 has only one serotype, EV-A, so it is a type A enterovirus (Chia et al., 2018). The incidence is cyclical, and the incubation period is not easily observable. Also, the incidence of EV71 severe disease is higher in areas with high temperature and humidity conditions (de Crom et al., 2016). As a critical neurotropic enterovirus, EV71 is occasionally associated with severe neurological disease and high mortality in infants and children (Guo et al., 2022). EV71 is a highly infectious RNA virus that induces type III interferon (IFN-*γ*) production after infection by stimulating the toll-like receptor 3/interferon regulatory factor 1 (TLR3/IRF1) signaling pathway (Su et al., 2020). Another study showed that the expression level of the inflammatory factor IFN-*α* was also significantly increased in the EV71-infected organism model (Wu, 2020). EV71 infection triggers a systemic inflammatory response, leading to an increased expression of tumor necrosis factor *α* (TNF-*α*), TNF-*β*, and other factors that promote inflammatory infiltration of cardiomyocytes (Ding et al., 2015; Wu, 2020), and the infection significantly promotes the expression of inflammatory factors such as interleukin 2 (IL-2) and IL-4 in thymocytes (Chung et al., 2008; Wu, 2020).

The EV71 genome is a single-stranded positive RNA molecule with an open reading frame (ORF) consisting of approximately 7,400 nucleotides (Plevka et al., 2012). The ORF can make the RNA encode multimeric precursor proteins, which can be further hydrolyzed into precursor proteins 1 (P1), P2, and P3, of which P1 can encode four viral capsid proteins (VPs). Most of the VPs exhibit viral antigen diversity due to exposure to the viral surface, and VP1 dominates this process (Cardosa et al., 2003). The presence of this diversity and changes in the viral spectrum due to the evolution and recombination of enteroviruses, among other factors (Zhang et al., 2015), make vaccines and drugs such as recombinant human IFN *α*-1b less active against EV71 and even produce adverse effects such as immune tolerance or cytotoxicity (Chen et al., 2008).

In addition to the variability of pathogens, another public health problem that cannot be ignored is drug resistance and adverse effects resulting from the overuse of anti-infective drugs (Dell’Annunziata et al., 2022). Natural drug extracts against viruses have many advantages, such as significantly reducing drug resistance in patients (Anand et al., 2019), lowering production costs, and being diverse biologically active compounds (Atanasov et al., 2015). Mosses are one of the oldest surviving land plants, and there are about 25,000 species of mosses in the world today, with at least 43 species having significant medicinal value (Asakawa et al., 2009). The subject of this experiment is BBS, a species of moss in the genus Bryophyta of the family *Bryum*, mainly distributed in tropical and warm temperate regions and parasitic on humid environments, decaying wood and humus at an altitude of nearly 1.5 km (Kunming Institute of Botany, Chinese Academy of Sciences, 2005). It has been reported that mosses are rich in terpenoids (Li et al., 2022), flavonoids (Hahn et al., 1995), alkaloids (Eichenberger et al., 1996), and coumarins (Brinkmeier et al., 1999), which may contribute to their pharmacological and biological activities.

Based on the above understanding, this experiment aimed to investigate the *in vitro* antiviral activity of the BBS extract against EV71, determine its active antiviral sites, and further investigate its modulating effect on the aforementioned cytokines through animal infection model tests. The monomers of the active sites were then separated and purified by preparative liquid chromatography (PLC). The monomers were subjected to *in vitro* antiviral assays to screen the best active monomers. Their chemical structures were analyzed by ultra-high performance liquid chromatography–mass spectrometry (UPLC-MS) and nuclear magnetic resonance (NMR). Finally, the antiviral activity of the monomers was verified by *in vitro* antiviral assays.

## 2 Materials and methods

### 2.1 Plant material

The experimental botanicals were purchased from the Xiangxi Tujia and Miao Autonomous Prefecture herb market in Hunan, China, and identified as the dried whole herb of BBS. The herbs were washed, dried, crushed using a mechanical grinder (Royalstar, Anhui, China), and passed through a sieve with an aperture of 0.25 mm.

### 2.2 Herbal extracts

The powdered BBS (180.0 g) was extracted by heating reflux with ultrapure water (2 × 1,800 mL), and the alcohol content of the solution was adjusted to 75% with 95% ethanol and left for 48 h at 4 C. The supernatant was extracted by filtration, centrifuged (3,000 rpm, 15 min), and concentrated under reduced pressure at 40 C. The concentrated solution was lyophilized to obtain 15.98 g of powder (yield 8.88%). The resulting lyophilized powder was divided equally into two parts and purified by silica gel column chromatography (Table 1) and polyamide column chromatography (Table 2). The eluted fractions of the silica gel column chromatography were monitored by a thin layer of silica gel (Table 3). The final homogeneous spot fractions obtained by silica gel thin-layer chromatography were numbered S1–S12, and the eluted fractions obtained by polyamide column chromatography were numbered P1–P6. *In vitro* and *in vivo* antiviral assays were performed on each elution site of S1–S12 and P1–P6 to determine the best antiviral activities of BBS.

**TABLE 1:** Composition of silica gel column chromatography eluent and its ratio.

No.	Solvent composition	Proportion
1	Dichloromethane:methanol	100:0
2		100:5
3		100:10
4		100:15
5		100:20
6		100:25
7		100:35
8		100:50
9		100:65
10		100:80
11		100:100
12		0:100

**TABLE 2:** Composition of polyamide column chromatography eluent and its ratio.

No.	Solvent combination	Proportion
1	Ultrapure water:ethyl alcohol	100:0
2		90:10
3		70:30
4		50:50
5		30:70
6		5:95

**TABLE 3:** Details of silica gel thin-layer chromatography monitoring.

No.	Solvent combinations	Proportion
1	Petroleum ether:ethyl acetate:methanol	6:1:1
2	Petroleum ether:ethyl acetate:methanol	6:2:1
3	Methanol:ethyl acetate:methylene chloride	1:1:3
4	Methanol:ethyl acetate:methylene chloride	1:1:1
5	n-Butanol:ethyl acetate:ultrapure water	7:2:1

### 2.3 Cells and viruses

Heteroploid Vero cells obtained from the African green monkey renal epithelial cell line of primates were provided by the Institute of Microbiology, Shandong First Medical University. The cells were cultured in 1640 medium (RPMI; Gibco; Thermo Fisher Scientific, Waltham, MA, United States) containing 10% fetal bovine serum (FBS; Biological Industries, Kibbutz Beit HaEmek, Israel) and 1% of penicillin–streptomycin antibacterial solution (Solarbio; Beijing Solarbio Science & Technology Co., Ltd., Beijing, China) and further incubated at 37 C with 5% CO_2_ at appropriate humidity conditions.

The preliminary study reported by Wu showed an antiviral effect of BBS on EV71 when compared with the effect on HSV-1, RSV, and H1N1 (Wu, 2020). In this study, the EV71 virus strain used was obtained from the Influenza Virus Research Laboratory, Institute of Viral Diseases, Chinese Center for Disease Control and Prevention. The EV71 virus solution (150 μL) was inoculated on a monolayer of Vero cells spread over the cell culture flask, and 6 mL of 2% cell maintenance solution was added and incubated at 37 C and 5% CO_2_ (Lishen Scientific Equipment Co., Ltd., Shanghai, China). The control were cells without the inoculated virus. The culture was terminated when the cytopathic effect (CPE) of the virus-added cells was ≥90% and still attached to the bottom of the culture flask. The virus was collected and transferred to a sterile clean conical tube after triple-freezing and triple-thawing at −80°C and 4°C and then centrifuged at 1,000 rpm for 5 min. The supernatant was taken for the experiment, while the precipitated part was kept as virus seeds.

### 2.4 Virus virulence assay

The collected virus supernatant was diluted at a ten-fold ratio to obtain 12 different concentrations of virus solution (10–10^−11^), and 100 μL of each was inoculated sequentially from left to right in a 96-well plate full of monolayer Vero cells. Each concentration was repeated thrice, while the cell control groups (100 μL of 2% cell maintenance solution) were set up and incubated for 48 h at 37 C and 5% CO_2_ under pre-established humidity conditions. The culture was terminated by washing with 100 μL of PBS (Biological Industries, Kibbutz Beit HaEmek, Israel) buffer and stained with 70 μL of 3-(4,5-dimethylthiazol-2-yl)-2,5-diphenyltetrazolium bromide (MTT) solution for 3 h under light-proof condition. Then, MTT was removed, and 100 μL of DMSO was used to dissolve formazan crystals for decolorization. The 96-well plate was placed in a gas-bath thermostatic shaker for 15 min and then in an enzyme standardizer (BioTek Instruments Inc., Highland Park, United States). The optical density (OD) values were measured at 490 nm. The half tissue culture infective dose (TCID_50_) of EV71 was calculated using the Reed–Muench equation:

$$\%Cell\;survival=\frac{{OD}_{490\;nm}\;\left(virus\right)}{{OD}_{490\;nm}\;\left(Cell\;control\right)}.$$

%Cytopathy=1−%Cell survival.

$$pd=\frac{p_{1}-50\%}{p_{1}-p_{2}}.$$

$${TCID}_{50}=Antilog\left(\log C_{1}+pd\times \log C_{m}\right).$$

Note: pd is the cytometric distance; p_1_ is the percentage of the cytopathic rate above 50%; p_2_ is the percentage of the cytopathic rate below 50%; C_1_ is the dilution of the viral solution when the cytopathic rate is above 50%; C_m_ is the multiplicative dilution factor.

### 2.5 Cytotoxicity assay

The purified fractions were dissolved in the 2% cell maintenance solution, and the samples were prepared at a concentration of 50 mg/mL. The samples were filtered through the 0.22-μm sterile microporous membrane to remove impurities and were diluted two-fold (2–2^−11^), and 100 μL of each was added to 96-well plates. Each concentration was tested thrice, and the cell control group, virus control group, and ribavirin control group (50 mg/mL, production lot number: 2104030641, Chenxin Pharmaceutical Co., Ltd., Shandong, China) were set up. The half-toxic concentration (TC_50_) of the drugs was calculated:

$${TC}_{50}=\left[Antilog\left(\log C_{2}-pd\right)\right]\times C_{0}.$$

Note: C_2_ is the drug dilution when the cytopathic rate is higher than 50%; C_0_ is the drug concentration in the first well of the 96-well plate.

### 2.6 Antiviral activity

The maximum non-toxic concentration of the purification sites was selected as the initial concentration based on the TC_50_ calculation. Then, 50 μL of each site diluted at a two-fold ratio was inoculated in the 96-well plates full of Vero cells, and 50 μL of EV71 solution was added. Each concentration was tested thrice. Afterward, the half-effective concentration (EC_50_) and therapeutic index (TI) of the drug at 490 nm were calculated according to the Reed–Muench method:

$${EC}_{50}=\left[Antilog\left(\log C_{3}-pd\right)\right]\times {C_{0}}^{\prime }.$$

$$TI=\frac{{TC}_{50}}{{EC}_{50}}.$$

Note: C_3_ is the drug dilution when cell viability is higher than 50%; C_0_’ is the drug concentration of the first well in the 96-well plate.

### 2.7 Time-of-addition determination

The 96-well plates full of monolayer Vero cells were inoculated with 100TCID_50_ of EV71 solution and treated with 50 μL S12 at pre-dose (−2 h), 0, 2, 4, 6, 8, 10, and 12 h of infection. The TI values of the live cells were measured by the aforementioned method.

### 2.8 Effective stage assay

To determine the *in vitro* antiviral mechanism of S12, we designed experiments with different dosing times using 96-well plates filled with Vero cells. Four experimental groups were designed, and each was repeated thrice (Liang et al., 2021).

(i) Direct killing: S12 was mixed with 100TCID_50_ virus solution at 100:1 (v/v) and incubated at 37°C for 2 h. Afterward, the mixture was added to 96-well plates. Cell viability was determined after 2 days, as described previously.
(ii) Inhibiting adsorption: S12 was first added to 96-well plates and incubated at 37°C–5% CO_2_ for 2 h before adding 100TCID_50_ EV71 solution to each well. Cell survival was determined after 48 h of incubation, as described previously.
(iii) Blocking proliferation: S12 was added after adding 100TCID_50_ virus solution and adsorbing at 37°C–5% CO_2_ for 2 h. Cell viability was determined after 2 days of cultivation.
(iv) Activating immunity: An equal volume of 100TCID_50_ virus solution was added immediately after the addition of S12, and cell survival was measured after 2 days.

### 2.9 *In vivo* antiviral activity

This experiment followed the method of previous experiments (Geng et al., 2019). A total of 60 3-weeks-old Balb/c mice were randomly divided into six groups. The daily doses used in the experiment were calculated by combining the results of S12 with ribavirin at a half-lethal dose (LD_50_) and the human–rat equivalent dose conversion formula. The mice in all groups except the blank control group were treated with 50 μL of EV71 solution for 2 days by intraperitoneal injection. The body weight of each mouse was recorded at 9:00 a.m. daily during the experiment. The doses and routes of administration used for the experiment are shown in Table 4.

**TABLE 4:** Details on dosage of administration.

Groups	Number	Virus infection	Drugs and dosage
Blank control group	10	No	Ultrapure water; 10.0 mL/kg/d
EV71 model group	10	Yes	—
Positive control group	10	Yes	Ribavirin; 100.0 mg/kg/d
High-dose group	10	Yes	S12; 4 g/kg/d
Middle-dose group	10	Yes	S12; 2 g/kg/d
Low-dose group	10	Yes	S12; 1 g/kg/d

### 2.10 Lung index inhibition ratio and histological analysis

The virus-infected mice were euthanized after treatment (5 days) with S12 under ether anesthesia by cervical dislocation by following the regulations and ethics of the use of experimental animals. The lung, skeletal muscle, and brain tissues were excised under aseptic conditions. To determine their lung index and lung index inhibition rate, paraffin sections of the left lung tissue from all mice were stained with hematoxylin and eosin (H&E) solution and then placed under a light microscope (Eclipse Ci-L, Nikon, Tokyo, Japan) for image scanning and acquisition with the aid of a panoramic scanner (PANNORAMIC Desk, 3DHISTECH, Budapest, Hungary).

$$lung\;index=\frac{wet\;weight\;of\;lung}{body\;weight}\times 100\%.$$

$$\%lung\;index\;inhibition=\frac{lung\;weight\;of\;model\;group-lung\;weight\;of\;experiment\;group}{lung\;weight\;of\;model\;group-lung\;weight\;of\;blank\;control\;group}\times 100\%.$$

### 2.11 Determination of viral titers in lung tissue

All right-sided lung tissues obtained after dissection were processed in a KZ-II tissue homogenizer (Servicebio, Hubei, China), mixed with 2% cell maintenance solution, and then centrifuged (2000 rpm; 15 min) to obtain the supernatant. They were inoculated sequentially in 96-well plates filled with monolayer Vero cells at two-fold dilution for 48 h and then treated by the MTT method to compare the virus titers in each group of lung tissues.

### 2.12 Identification of *in vivo* antiviral mechanisms

This work aims to study the effect of S12 on regulating serum cytokines in EV71-infected mice and its effect on gene expression in the lung tissue. The blood of each group of mice was centrifuged at 3,000 rpm for 20 min, and the serum was collected. The effects of S12 on the expression levels of IFN-*γ*, TNF-*α*, IL-2, and nuclear factor kappa-B (NF-*κ*B) (p65) in the serum were determined by enzyme-linked immunosorbent assay (materials were purchased from Servicebio, Hubei, China). Then, 100 μL of the serum samples were taken in 96-well plates, and blank control groups were set and incubated at 37 °C for 2 h. The horseradish peroxidase–labeled streptavidin was diluted at 1:100, and 100 μL of each was placed in each well of the 96-well plates, sealed, and incubated for 30 min at 37 °C in the dark after constant shaking at 300 rpm. After the reaction, 100 μL of TMB (3′, 3′, 5′, 5′-tetramethylbenzidine) was added to each well under light-proof conditions for 30 min, and the reaction was terminated with 100 μL of sulfuric acid to detect the solution’s OD_450nm_ and OD_570nm_, and the calibration value = OD_450nm_ − OD_570nm_.

Total RNA was extracted from mouse lung tissues using TRIzol reagent (Servicebio, Hubei, China) in tissue homogenates, followed by qRT-PCR analysis of three target genes (EV71, TLR3, and TLR4). Equal volumes of Oligo (dT) 18 primer and random hexamer primer were mixed with RNA solution at 1 μL:2 μg dilution and incubated for 5 min at 65 °C. The cDNA was synthesized by reverse transcription with Servicebio^®^ RT First Strand cDNA Synthesis Kit (Servicebio, Hubei, China) and SYBR Green qPCR Master Mix (2×) (Low ROX), and the reaction solution was incubated at 42 °C for 60 min and then at 70 °C for 5 min.

In addition, 60 3-weeks-old Balb/c mice were randomly divided equally into six groups, and the daily dose of the Janus kinase 2 (JAK2)–specific inhibitor AG490 (Merck, Germany; CAS: 133,550-30-8) was referenced to the available research (Wu, 2012). The mice in the blank control group and AG490 group were administered intraperitoneal injections of 50 μL saline for 2 consecutive days, and others were administered equal amounts of EV71. The specific mode of administration from days 2–8 is shown in Table 5. Lung tissues were taken as described in Section 2.12 and assayed for *Homo sapiens* signal transducer and activator of transcription 1 (STAT1) expression with qRT-PCR. The details on the specific primers used for the analysis of cytokines and genes are shown in Supplementary Appendix S1. The data were processed using the 2^−ΔΔCT^ (Livak) method:

$${∆CT}_{test}={CT}_{target,test}-{CT}_{ref,test}.$$

$${∆CT}_{tecalibratorst}={CT}_{target,calibrator}-{CT}_{ref,calibrator}.$$

$$∆∆CT={∆CT}_{test}-{∆CT}_{tecalibratorst}.$$

$$\%expression\;level=2^{-∆∆CT}.$$

**TABLE 5:** Details on dosage of administration (STAT1 mRNA groups).

Group	Number	Virus infection	Drugs and dosage
(A) Blank control group	10	No	50 μL/d saline
(B) EV71 model group	10	Yes	50 μL/d saline
(C) AG490 group	10	No	10 mg/kg/d AG490
(D) EV71 + middle-dose group	10	Yes	2 g/kg/d S12
(E) EV71 + AG490 group	10	Yes	10 mg/kg/d AG490
(F) EV71 + AG490 + middle-dose group	10	Yes	2 g/kg/d S12 + 10 mg/kg/d AG490

AG490: dissolve 10 mg of AG490 standard in 9 mL of DMSO and dilute it to 45% by volume in saline.

For the expression of TLR3 and TLR4, in order to further investigate the specific regulatory effect of BBS on the upstream and downstream factors of TLR3 and TLR4, this experiment was specially supplemented with the study of the effect of TLR3 and TLR4 agonists on BBS-mediated induction of inflammation against EV71. A total of 70 3-weeks-old Balb/c mice were randomly divided into seven groups, and all groups except the blank and agonist groups were administered polyinosinic:polycytidylic acid (Poly 1:C; TLR3 agonist; Sigma, America) at a dose of 5 μg/kg/d (Wang et al., 2020) and monophosphoryl lipid A (MPLA; 1 μg/mL, TLR4 agonist; Sigma, America) at a dose of 1 μg/d (Liao, 2020) for five consecutive days after two consecutive days of intraperitoneal injections of 50 μL of EV71 except for the blank group. The specific mode of administration and dosage is shown in Table 6.

**TABLE 6:** Details on dosage of administration in TLR3 and TLR4 agonist groups.

Groups	Number	Virus infection	Drugs and dosage
Blank control group	10	No	10.0 mL/kg/d ultrapure water
Agonist group	10	No	5 μg/kg/d Poly (1:C) + 1 μg/d MPLA
EV71 + agonist group	10	Yes	5 μg/kg/d Poly (1:C) + 1 μg/d MPLA
Positive + agonist group	10	Yes	100.0 mg/kg/d ribavirin + 5 μg/kg/d Poly (1:C) + 1 μg/d MPLA
High-dose + agonist group	10	Yes	4 g/kg/d S12 + 5 μg/kg/d Poly (1:C) + 1 μg/d MPLA
Middle-dose + agonist group	10	Yes	2 g/kg/d S12 + 5 μg/kg/d Poly (1:C) + 1 μg/d MPLA
Low-dose + agonist group	10	Yes	1 g/kg/d S12 + 5 μg/kg/d Poly (1:C) + 1 μg/d MPLA

### 2.13 Isolation of active monomers and determination of their chemical structures

By using a UV spectrophotometer, S11 and S12 were scanned at full wavelength and their absorption was measured at 260 nm. Gradient elution was performed on the PLC on the Eclipse XDB-C_18_ column (4.6 × 250 mm, 5 μm) with a mobile phase of 0.1% phosphoric acid (A):acetonitrile (B) at 260 nm and a flow rate of 1.0 mL/min. The purity of each monomer (C1–C5) was determined using HPLC on the ODS-2 column (10 × 250 mm, 10 μm) with a mobile phase of ultrapure water (A):acetonitrile (B) = 20:80 and a flow rate of 1.0 mL/min. The active monomers were then screened by *in vitro* antiviral assays. Finally, the chemical structures of the optimal active monomer were obtained by physicochemical identification and UPLC-MS combined with NMR. The conditions are as follows:

Chromatographic conditions: Hypersil GOLD AQ column (100 × 2.1 mm), mobile phase: ultrapure water (A):acetonitrile (B) = 5:95, detection wavelength: 260 nm, flow rate: 0.2 mL/min, injection volume: 20 μL, and column temperature: 30 °C.

Mass spectrometric conditions: ionic sources: electrospray ion source (ESI), dry gas flow rate: 35 L/min, dry gas temperature: 350 °C, and mass spectrometry scanning range: 100–2,000 m/z.

The results of the chemical structure analysis of monomers C3 and C4 are as follows:

ESI-MS *m*/*z*: 595 [M + H]^+^, 593 [M-H]^−^;

^1^H-NMR (400 MHz, DMSO-*d*6) *δ*: 13.53 (1H, s, 5-OH), 10.42 (1H, brs, 4′-OH), 7.96(2H, d, *J* = 8.8 Hz, H-2′, 6′), 6.94 (2H, d, *J* = 8.8 Hz, H-3′, 5′), 6.91 (1H, s, H-8), 6.88 (1H, s, H-3), 4.73(1H, d, *J* = 10.1 Hz, H-1″), 4.98 (1H, d, *J* = 7.0 Hz, H-1‴);

^13^C-NMR (100 MHz, DMSO-*d*6) *δ*: 164.2 (C-2), 103.2 (C-3), 182.4 (C-4), 159.3(C-5), 110.6 (C-6), 162.5 (C-7), 93.7 (C-8), 156.4 (C-9), 104.9 (C-10), 120.9 (C-1′), 128.6 (C-2′, 6′), 116.0 (C-3′, 5′), 161.4 (C-4′), 101.2 (C-1‴), 81.5 (C-5″), 78.9(C-5‴), 77.2 (C-3″), 75.8 (C-3‴), 73.8 (C-1″), 72.7 (C-2‴), 70.9 (C-2″), 69.6 (C-4‴), 69.5 (C-4″), 60.7 (C-6‴), 60.6(C-6″).

### 2.14 *In vitro* antiviral effect of active monomer

Saponarin standard (Ku’er, Anhui, China; CAS: 8047-15-2) at 1, 2, 5, 10, and 50 mg/mL concentrations were prepared and subjected to *in vitro* antiviral assays as described in Section 2.6. The TI values of saponarin were calculated separately for each concentration condition to verify and determine its optimal *in vitro* antiviral concentration.

### 2.15 Statistical analysis

The normality was tested through the Shapiro–Wilk test, and then multiple comparisons were performed by using ANOVA. All data were processed using IBM SPSS 26 (Armonk, United States).

## 3 Results and discussion

### 3.1 TCID_50_ value of EV71; TC_50_, EC_50_, and TI values of purification sites and ribavirin

The TCID_50_ value of EV71 was calculated from the formula as 10^−3.256^. The higher the TC_50_ value, the better the drug’s safety, and the lower the EC_50_ value, the more effective the drug is, so a higher TI value indicates that the drug is more effective. According to the results (Supplementary Appendix S2), it was observed that the *in vitro* antiviral activity of S12 (TI = 82.701) was significantly better than that of ribavirin (TI = 70.132), and there was no antiviral effect in other fractions except S10–S12 (TI_S10_ = 19.922, TI_S11_ = 42.817).

### 3.2 Time-of-addition determination

**FIGURE 1:**
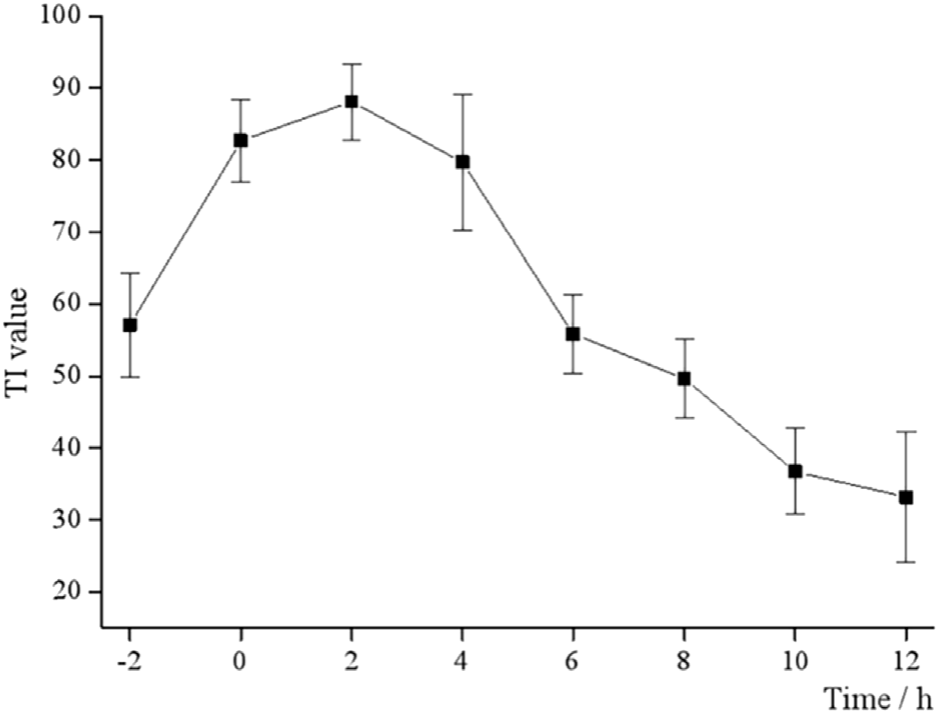
TI values at different time points.

EV71 has a certain “escape” phenomenon during the infection process in the organism, which is the vulnerability of its infectivity and the key to studying its antiviral drugs (Zhou et al., 2022). Different dosing time tests were designed to determine the antiviral activity of BBS, i.e., 50 μL S12 was added at each of the eight time points before and after the Vero cells were infected with EV71. As shown in Figure 1, the TI value at 0 h is the closest to the TI value at 4 h, and the TI value at −2 h is the closest to the TI value at 6 h and reaches the peak TI value at 2 h. This result also suggested that the antiviral activity of S12 showed a parabolic relationship with the dosing time, i.e., S12 administration within 0–6 h after viral infection significantly inhibited virus proliferation. Hence, the aforementioned results also imply that BBS can exert antiviral activity by activating the body’s immune function and affecting the proliferation cycle of the virus.

### 3.3 Effective stage analysis

The effective phase analysis assays were designed to explore the antiviral activity phase of BBS against EV71. The mode of action was determined by measuring the TI values of each reaction phase. The TI_i_, TI_ii_, TI_iii_, and TI_iv_ were determined to be 38.714, 58.334, 88.361, and 83.012, respectively. The analysis revealed that the antiviral effects of (i) and (ii) groups were significantly lower than those of (iii) and (iv) groups, indicating that the BBS antiviral activity may be due to different pathways, such as blocking the proliferation of the virus in cells and activating the immune response against the viral infection. In addition, BBS can also directly kill viruses and inhibit the adsorption and invasion of viruses into cells to some extent. Nonetheless, the specific mechanism of the antiviral activity of BBS against EV71 remains unknown.

### 3.4 *In vivo* anti-EV71 activity

**FIGURE 2:**
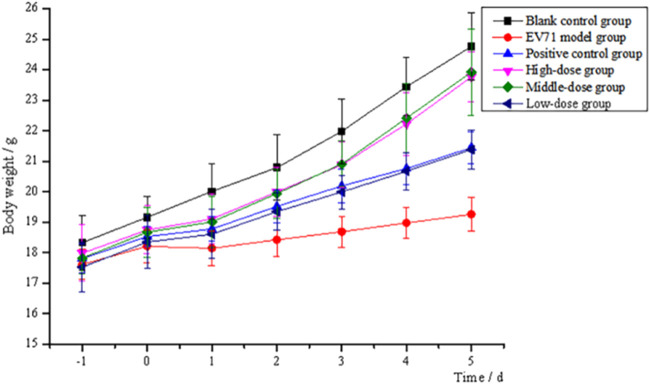
Trend of body weight changes of mice (high-dose: 4 g/kg/d; middle-dose: 2 g/kg/d; low-dose: 1 g/kg/d).

After continuous intraperitoneal injection of EV71 virus solution in mice for 2 days, all groups of mice except the blank control group showed significant signs of infection, such as significant reduction in diet and water intake, slow weight gain, significant slow movement, rapid respiration, elevated body temperature, and knotted and curled body hair with no luster. All mice treated with the administration of S12 showed significantly reduced infection characteristics when compared to the EV71 model group mice. The body weight gain in the blank control group mice was stable throughout the process. S12 in the high-dose (4 g/kg/d) and middle-dose (2 g/kg/d) groups presented an average body weight gain starting on day 4 of administration. By contrast, the body weight gain in mice in the ribavirin and low-dose (1 g/kg/d) groups remained slow (Figure 2). The following results indicate that the therapeutic effect of BBS on EV71-infected mice shows significant dose dependence.

### 3.5 Lung index inhibition ratio and histological analysis

**FIGURE 3:**
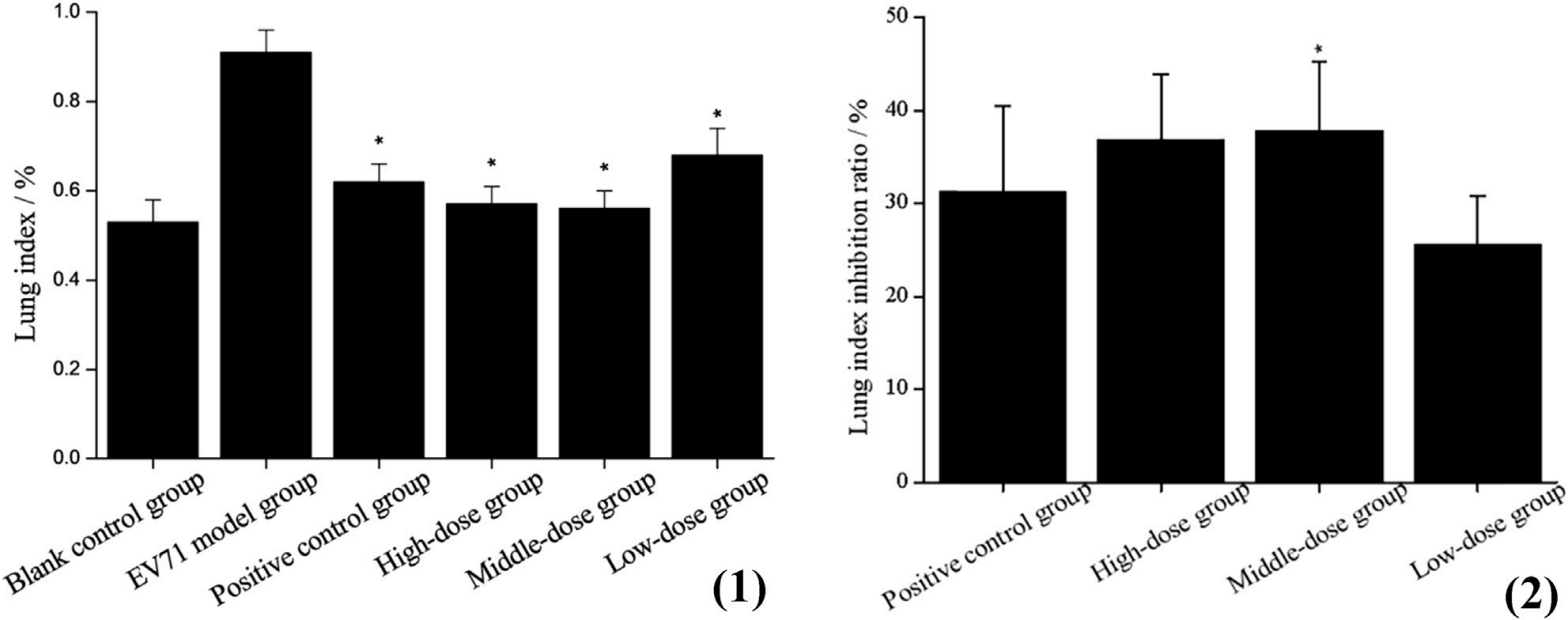
(1) Lung index (the results are presented as mean ± SD, N = 5; *p < 0.05 compared with EV71 model group). (2) Lung index inhibition ratio (the results are presented as mean ± SD, N = 5; *p < 0.05 when compared with the positive control group). Note: high-dose: 4 g/kg/d; middle-dose: 2 g/kg/d; low-dose: 1 g/kg/d.

The determination of the lung index for each group of mice revealed that the model group presented a higher lung index than the blank group, which indicates that EV71 infection causes swelling, congestion, hyperplasia, and hypertrophy of lung tissue, leading to increased lung tissue weight. The lung index was somewhat inhibited after the administration of S12, and the high-dose (4 g/kg/d) and middle-dose (2 g/kg/d) groups were significantly better than the positive control group (Figure 3). These results strongly suggest that S12 can reduce lung injury caused by EV71.

**FIGURE 4:**
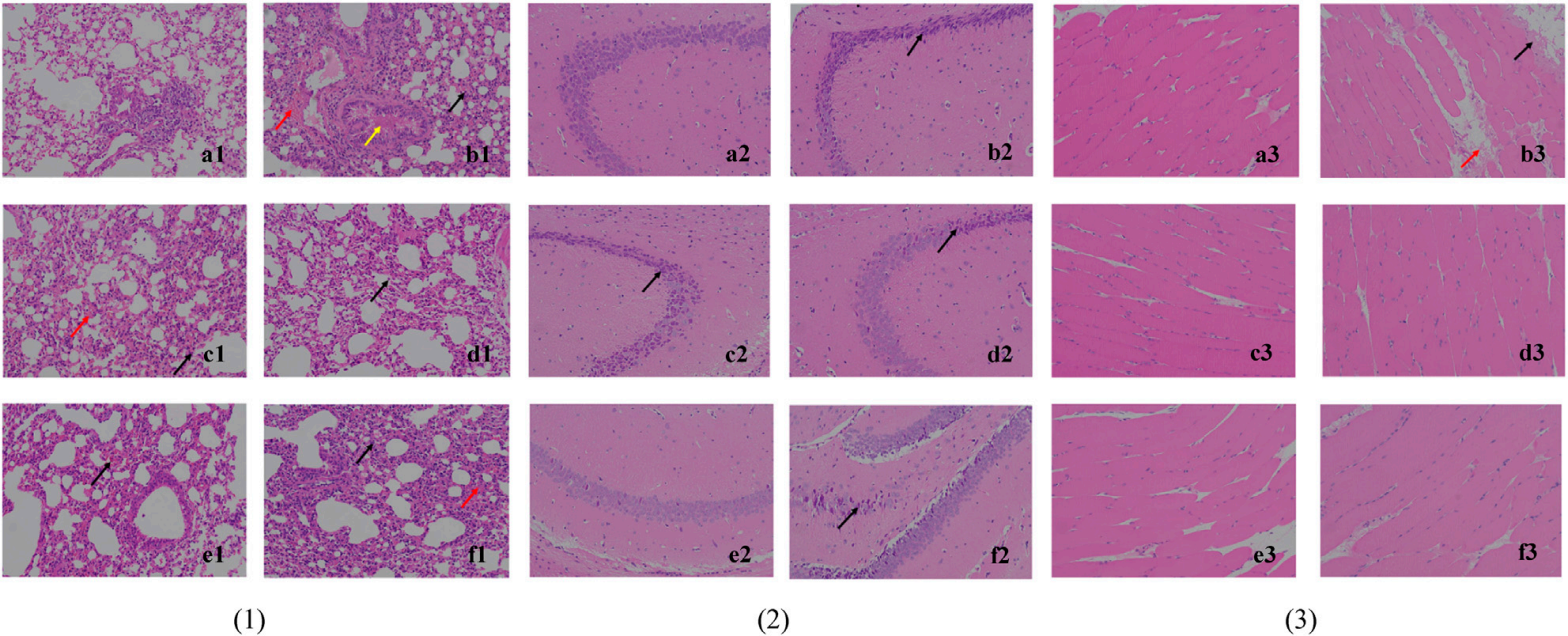
Histological observation of lung tissue (1), hippocampus CA3 (2) and skeletal muscle tissue (3) (200×). Note: (a) blank control group; (b) EV71 model group; (c) positive control group; (d) high-dose group (4 g/kg/d); (e) middle-dose group (2 g/kg/d); (f) low-dose group (1 g/kg/d).

The histological observation of lung tissues is shown in Figure 4, (1). The alveolar walls of all mice except the blank group are thickened and the thickening is the lowest in the high-dose (4 g/kg/d) and middle-dose (2 g/kg/d) groups: (b1) monocytes and granulocytes are infiltrated (black arrows), some capillaries show blood pooling (red arrows), and the bronchi are filled with eosinophilic secretions (yellow arrows). The treatment groups (c1), (d1), and (e1) present a lower degree of lung tissue lesions, while infiltration (black arrow), bruising (red arrow), and compensatory enlargement of alveoli are seen in (f1). This could indicate that S12 potentially has a dose-dependent protective effect against lung injury caused by EV71.

The histological observation of brain tissues is shown in Figure 4, (2). Through the analysis and processing of the brain tissue, it is found that EV71 perhaps has a more significant effect on the hippocampus CA3 of mice, which can provide a certain reference value for subsequent related research. In the blank control group (a2), the cone cells are seen regularly arranged, the nucleus and cytoplasm are restricted, the nucleus is large and round, the nucleolus is prominent, and the cell shape is regular. In (b2), (c2), (d2), and (f2), the hippocampal cone cells are found to be solidly shrunken, deeply dyed, and irregularly arranged (black arrows), and the nuclei are not restricted from the cytoplasm. By contrast, the mice in the middle-dose (2 g/kg/d) group (e2) have the lowest degree of brain tissue lesions, thus indicating to the most significant protective effect against brain injury caused by EV71.

The histological observation of skeletal muscle tissue is shown in Figure 4, (3). The skeletal muscle tissue of the model group (b3) mice show marginal muscle fiber lysis, nucleus fragmentation, disorganized and eosinophilic homogeneous or flocculent cell division (black arrows), and some eosinophilic flocculent material (red arrows) in the interstitium of muscle fibers. The aforementioned symptoms are improved in all dosing groups, indicating that the efficacy was not dose dependent.

### 3.6 Measurement of viral titers in lung tissue

**FIGURE 5:**
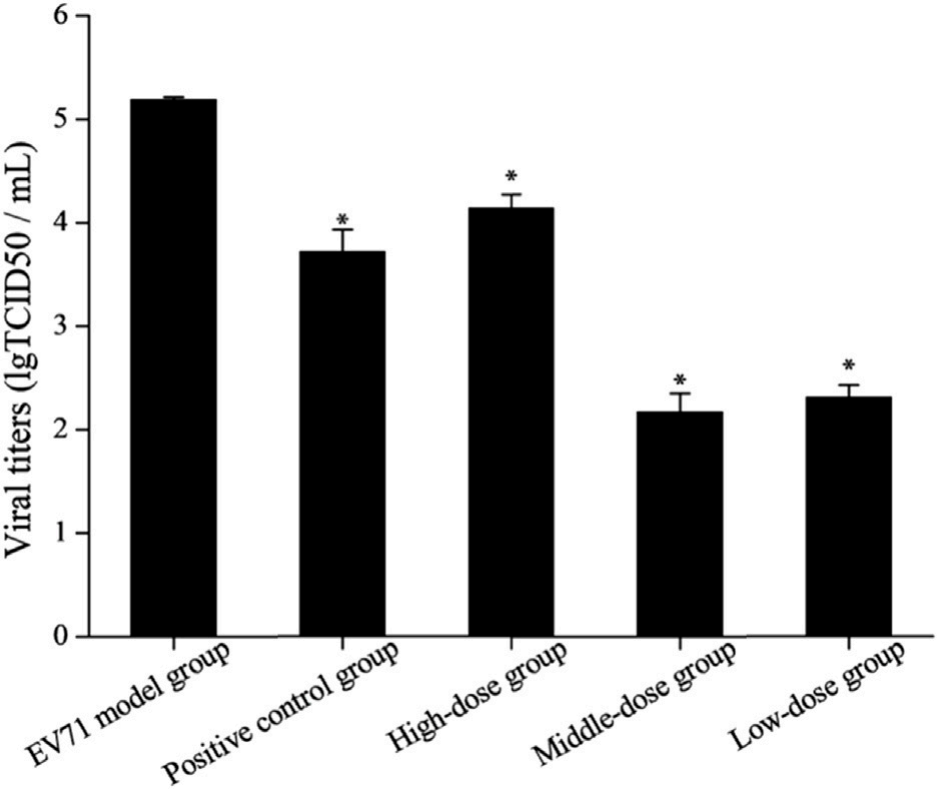
Results of viral titers in mice lung tissue (the results are presented as mean ± SD, N = 5; *p < 0.05 compared with the EV71 model group). Note: high-dose: 4 g/kg/d; middle-dose: 2 g/kg/d; low-dose: 1 g/kg/d.

Due to the dose-dependent nature of antiviral drugs in their efficacy studies and the close relationship between their efficacy and viral titer, accurate quantitative analysis of viruses has become increasingly indispensable (Guo et al., 2023). It is found by observing the cells daily that the virus titer reached a maximum on day 7 after the lesion, so the lung tissue on day 7 was used for testing and comparison in this study (Figure 5). The results showed that the viral titers in the lung tissues of the mice in each drug group were reduced to some extent when compared with the model group (*p* < 0.05). The viral titers of mice in the middle-dose (2 g/kg/d) and low-dose (1 g/kg/d) groups were significantly lower than those in the positive control group, indicating that the inhibitory effect of S12 on EV71 viral titers showed significant dose dependence and demonstrating that S12 could significantly inhibit the proliferation of EV71 in lung tissues and alleviate the lung lesions caused by it.

### 3.7 Identification of *in vivo* antiviral mechanism

**FIGURE 6:**
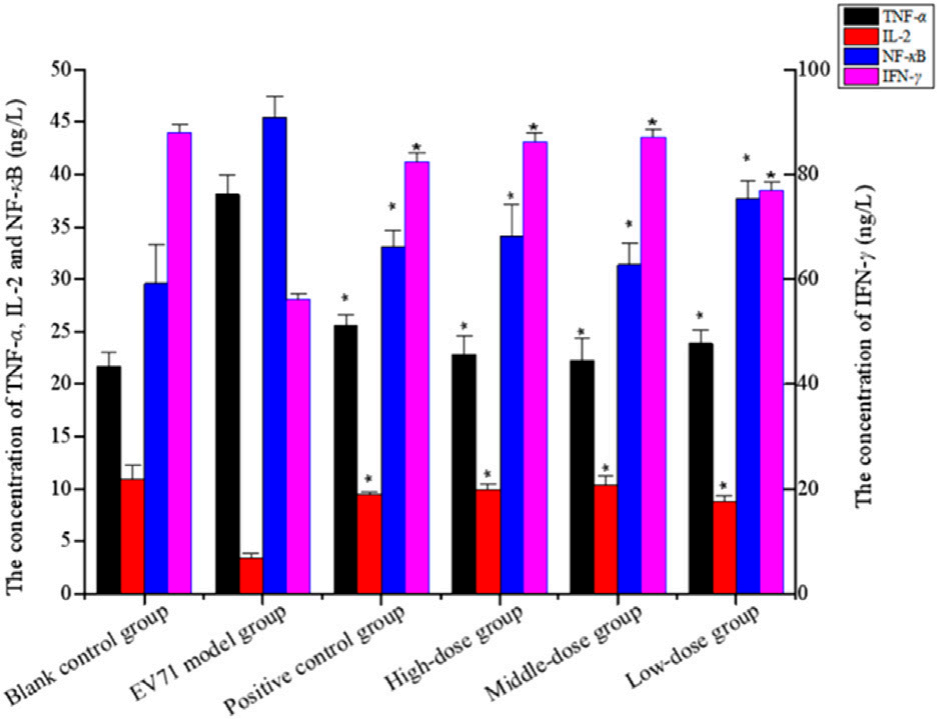
Concentration of TNF-*α*, IL-2, NF-*κ*B, and IFN-*γ* (the results are presented as mean ± SD, N = 5; *p < 0.05 compared with the EV71 model group). Note: high-dose: 4 g/kg/d; middle-dose: 2 g/kg/d; low-dose: 1 g/kg/d.

Studies have shown that NF-*κ*B, IFN-*γ*, and IL-2 are essential cytokines for improving immune function, and effective phase analysis experiments suggest that BBS can present antiviral effects by enhancing immune function. As shown in Figure 6 (Supplementary Appendix S3), the treatment with S12 for 7 days inhibited the expression of TNF-*α* while increasing the expression levels of NF-*κ*B, IL-2, and IFN-*γ*, suggesting that S12 could indirectly exhibit antiviral activity by alleviating the inflammatory response caused by EV71 infection. Among them, the high-dose (4 g/kg/d) and middle-dose (2 g/kg/d) groups showed remarkable antiviral activity due to better modulation of the aforementioned factors (NF-*κ*B, IFN-*γ*, IL-2, and TNF-*α*) than the ribavirin group after administration.

Since IFN has been revealed to exhibit impressive effects in various aspects such as the antiviral effect, the JAK/STAT signaling pathway has gradually become a major hotspot in order to investigate how the body responds to IFN to generate its antiviral mechanism. Activation of the JAK/STAT pathway provides a profound defensive self-protection effect to exert multiple antiviral activities (Philips et al., 2022). The relative expression of STAT1 in the lung tissues is shown in Table 7. The mice in group E died from day 2 onward, mice in groups C and F died from day 3 onward, and mice in group B died from day 6 onward, but mice in groups A and D all survived. The relative expression of STAT1 in groups C, E, and F did not significantly differ from that in group A but was significantly lower than that in groups B and D. The results (Supplementary Appendix S4, Table 7) showed that EV71 significantly increased the expression of STAT1 in lung tissues, AG490 markedly inhibited this phenomenon, and S12 can activate the JAK2/STAT1 signaling pathway by promoting the expression of IFN-*γ* to induce innate immune activity against EV71.

**TABLE 7:** Relative expression of STAT1 mRNA in lung tissues.

Group	Relative expression
(A) Blank control group	1.00 ± 0.00
(B) EV71 model group	7.81 ± 0.50**
(C) AG490 group	0.42 ± 0.03
(D) EV71 + middle-dose group	9.59 ± 0.38**
(E) EV71 + AG490 group	0.35 ± 0.44
(F) EV71 + AG490 + middle-dose group	0.35 ± 0.18

The results are presented as mean ± SD, N = 5; **p < 0.01 compared with the blank control group.

**FIGURE 7:**
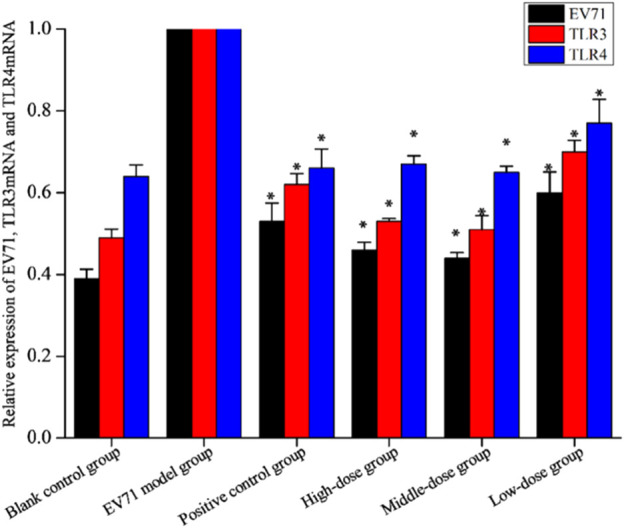
Relative expression of EV71, TLR3, and TLR4 (the results are presented as mean ± SD, N = 5; *p < 0.05 compared with the EV71 model group). Note: high-dose: 4 g/kg/d; middle-dose: 2 g/kg/d; low-dose: 1 g/kg/d.

TLRs are essential for the infected body to perform functions such as immune activation. In this experiment, the EV71 viral load in mouse lung tissues was further determined at the genetic level by qRT-PCR, and the expression levels of TLR3 and TLR4 were also examined. As shown in Figure 7, EV71 significantly upregulated the expression of TLR3 and TLR4 in the lung tissue of virus-infected mice. The use of ribavirin or S12 could interfere with this expression result to a certain extent, among which the effect of the high-dose (4 g/kg/d) and middle-dose (2 g/kg/d) groups was still more prominent. The regulatory effects on TLR3 and TLR4 suggest that S12 may express its antiviral activity mainly by acting on the viral attachment and proliferation phases. Figure 6 also reveals that the groups with higher activity actually achieved activity by mainly modulating NF-*κ*B, TNF-*α*, IFN-*γ*, and TLR3. The synergistic effect could enhance the host’s immune function and thereby the active substances’ antiviral activity (Chen et al., 2021).

Further results showed that the agonists of TLR3 and TLR4 added to the original *in vivo* pharmacodynamic experiments not only upregulated the expression of TLR3 and TLR4 but also significantly increased the expression of cytokines, such as TNF-*α*, IL-2, and IFN-*γ*. The specific degree of change in the expression of each factor is shown in Table 8. In the course of the experiment, the mice in the “EV71 model + agonist group” started to die from day 3 of drug administration, which was considered to have been caused by a high level of inflammation, resulting in severe damage. In addition, the data indicate that even though the addition of TLR3 and TLR4 agonists increased the expression of inflammatory factors, S12 could still express its regulatory effects on cytokines such as TLR3, TLR4, TNF-*α*, IL-2, and IFN-*γ*, and its effect was most prominent in the middle-dose (2 g/kg/d S12 + 5 μg/kg/d Poly (1:C) + 1 μg/d MPLA) group. The results of this experiment can further indicate that BBS can exert its antiviral effect by regulating the expression of the upstream and downstream cytokines of the aforementioned factors.

**TABLE 8:** Effect of S12 on various cytokines in the serum of various groups of mice before and after the addition of TLR3 and TLR4 agonists.

Groups	TLR3		TLR4		TNF-*α* (ng/L)		IL-2 (ng/L)		IFN-*γ* (ng/L)	
	Before	After	Before	After	Before	After	Before	After	Before	After
Blank control group	0.32 ± 0.05	0.34 ± 0.02	0.41 ± 0.02	0.45 ± 0.09	21.71 ± 1.26	120.43 ± 7.71	10.90 ± 1.36	293.43 ± 6.47	88.03 ± 1.54	161.10 ± 8.11
Agonist group	—	0.79 ± 0.04	—	0.81 ± 0.02	—	383.25 ± 4.01	—	77.24 ± 4.39	—	92.16 ± 6.06
EV71 model group	1.00 ± 0.00	1.00 ± 0.00	1.00 ± 0.00	1.00 ± 0.00	38.12 ± 1.79	435.87 ± 2.00	3.37 ± 0.53	68.72 ± 5.11	56.03 ± 1.15	58.81 ± 9.82
Positive control group	0.62 ± 0.03^*^	0.71 ± 0.03^**^	0.64 ± 0.05^*^	0.72 ± 0.04^*^	25.54 ± 1.09^*^	160.05 ± 9.57^**^	9.42 ± 0.33^*^	147.26 ± 2.39^**^	82.35 ± 1.79^*^	132.01 ± 8.00^*^
High-dose group	0.53 ± 0.01^*^	0.72 ± 0.07^*^	0.67 ± 0.02^*^	0.76 ± 0.04^*^	22.80 ± 1.79^*^	162.14 ± 3.61^**^	9.91 ± 0.53^*^	147.80 ± 3.98^**^	86.24 ± 1.69^*^	130.44 ± 3.31^**^
Middle-dose group	0.51 ± 0.03^*^	0.57 ± 0.02^**^	0.65 ± 0.02^*^	0.64 ± 0.05^**^	22.25 ± 2.09^*^	127.73 ± 7.42^**^	10.41 ± 0.82^*^	291.09 ± 1.86^**^	87.14 ± 1.51^*^	157.97 ± 5.64^**^
Low-dose group	0.70 ± 0.03^*^	0.79 ± 0.10	0.77 ± 0.06^*^	0.78 ± 0.02^*^	23.90 ± 1.26^*^	223.66 ± 6.60^**^	8.77 ± 0.63^*^	97.45 ± 5.73^**^	76.97 ± 1.51^*^	108.55 ± 7.98^*^

“Before” and “after” refer to the treatment of mice in the manner of administration shown in Table 4 and Table 6, respectively (the results are presented as mean ± SD, N = 5; *p < 0.05 compared with the EV71 model group, **p < 0.01 compared with the EV71 model group).

### 3.8 Isolation of active monomers and determination of their chemical structures

*In vitro* antiviral trials on the isolated monomers revealed that C3, C4, and C5 monomers were all more effective than Ribavirin (TI = 72.439, TC_50_ = 2^−0.772^, and EC_50_ = 2^−6.923^) with TI values of 92.092 (TC_50_ = 2^−0.193^ and EC_50_ = 2^−6.718^), 99.733 (TC_50_ = 2^−0.151^ and EC_50_ = 2^−6.791^), and 77.172 (TC_50_ = 2^−0.213^ and EC_50_ = 2^−6.483^), respectively. By qualitative analysis of these three effective monomers, the results of physicochemical identification of monomers C3 and C4 were positive for ferric trichloride reaction, hydrochloric acid–magnesium powder reaction, Gibb’s reaction, and Molish's reaction. The sample quantity of monomer C5 was too limited, so the exact information could not be measured. Still, the analysis of its liquid-mass and ^1^H-NMR could tentatively determine that it belongs to the glycosides (Supplementary Appendix S5).

From the review of the pertinent data (Supplementary Appendix S6), it was determined that the compound is a flavonoid–saponin (saponarin, CAS: 8047-15-2) with a molecular weight of 594.52 (Li et al., 2015). In some cases, since the ionic fragments of C3 are virtually identical to those of C4 and the sample sizes of C3 and C4 are minimal, it is presumed that the two are isomers of each other. The molecular formula is C_27_H_30_O_15_.

### 3.9 *In vitro* antiviral effect of active monomers

The findings of this experimental study show that the TI values of saponarin standard (in increasing order of concentration) are 26.649, 41.585, 105.859, 63.911, and 48.068, while the TI values of ribavirin and S12 are 71.407 and 80.449 (Supplementary Appendix S7), respectively. Therefore, it is presumed that 5 mg/mL is the optimal *in vitro* antiviral concentration of saponarin.

## 4 Conclusion

In conclusion, we confirm the anti-EV71 activity of BBS by *in vitro* and *in vivo* studies. The antiviral activity of the aqueous extract of BBS was found to be the strongest, in part obtained by silica gel column (dichloromethane:methanol = 0:100) separation and purification. BBS shows strong inhibitory activity in the middle and late stages of the EV71 activity cycle and could dramatically block the multiplication of the virus and activate the immune response of cells against viral infection, thus reducing the extent of EV71-induced pathologies in the lungs, brain, and skeletal muscle tissues. These findings fully justify the anti-EV71 activity of BBS. Further investigation established that BBS modulates the expression of upstream and downstream factors for combined antiviral effects by regulating the expression of TLR3, TLR4, TNF-*α*, IL-2, and IFN-*γ* by modulating the activation of NF-*κ*B and JAK2/STAT1 signaling pathways. In particular, it eliminates viruses directly and inhibits the adhesion and invasion of viruses into cells. Further characterization and authentication of S11 and S12 with techniques such as UPLC-MS and NMR disclosed that saponarin was one of the principal active singletons for BBS to exhibit antiviral effects. Recent studies have shown that the vaccines associated with HFMD can cause high fever, abdominal pain, and other adverse effects in children in clinical settings and are also expensive (Aswathyraj et al., 2016; Zhang et al., 2023). Natural medicines with anti-EV-71 activity may become the “stars of the future” for the prevention of HFMD because they are affordable and have remarkable characteristics such as low adverse effects and side-effects. As has been illustrated above, it can be concluded that BBS is a natural anti-EV71 product with great potential for development, and saponarin is its inescapable active monomer.

Nevertheless, since the sample size of C5 in this research was too weak to obtain accurate information, it should be further examined to elucidate the other antiviral constituents of BBS, especially the monomeric substances in the S12, which have the most striking anti-EV71 activity. Simultaneously, considering the safety and reliability of the product, toxicological and pharmacokinetic studies of BBS should be carried out, and an attempt should be made to develop an equivalent formulation of BBS on this basis to establish scientific support for its timely clinical application.

## Data Availability

The original contributions presented in the study are included in the article/Supplementary Material; further inquiries can be directed to the corresponding authors.

## Abbreviations

EV71, enterovirus; HFMD, hand, foot, and mouth disease; IFN-γ, type III interferon; TLR, toll-like receptor; IRF1, interferon regulatory factor 1; TNF, tumor necrosis factor; IL, interleukin; ORF, open reading frame; P1/2/3, proteins 1/2/3; VPs, viral capsid proteins; BBS, *Bryum billardieri* Schwaegr.; PLC, preparative liquid chromatography; UPLC-MS, ultra-high performance liquid chromatography–mass spectrometry; NMR, nuclear magnetic resonance; HSV, herpes simplex virus; RSV, respiratory syncytial virus; CPE, cytopathic effect; OD, optical density; TCID_50_, tissue culture infective dose; MTT, 3-(4,5-dimethylthiazol-2-yl)-2,5-diphenyltetrazolium bromide; TC_50_, half toxic concentration; EC_50_, half effective concentration; TI, therapeutic index; H&E, hematoxylin and eosin; LD_50_, half lethal dose; Poly (1:C), polyinosinic–polycytidylic acid; MPLA, monophosphoryl lipid A; JAK2, Janus kinase 2; STAT1, *Homo sapiens* signal transducer and activator of transcription 1; AG490, tyrphostin B42; NF-κB, nuclear factor kappa-B; HPLC, high-performance liquid chromatography.
