# Supplementary material for: Bryum billardieri Schwaegr. against EV71 infection: in vitro and in vivo antiviral effects, identification of molecular mechanisms and active monomers
Source: Front Pharmacol. 2023 Aug 15;14:1164784. doi: 10.3389/fphar.2023.1164784 (PMC10463183; doi:10.3389/fphar.2023.1164784)
Supplement: Supplementary file 1 [file Table1.DOCX]

**Supplementary materials**

**Appendix 1**. The details on the specific primers used for analysis of cytokines and genes.

| Target gene | Forward Primer | Reverse Primer | PrimerBank ID |
| --- | --- | --- | --- |
| TNF-*α* | 5'-CAGGCGGTGCCTATGTCTC-3' | 5'- CGATCACCCCGAAGTTCAGTAG-3' | 133892368c1 |
| IL-2 | 5'-ACACCTTTAATTGGTCAACACGA-3' | 5'- CCTGCTACGTTCTCTACCTCT-3' | 10181200a1 |
| IFN-*γ* | 5'-ATGAACGCTACACACTGCATC-3' | 5'-CCATCCTTTTGCCAGTTCCTC-3' | 33468859a1 |
| NF-*κ*B (p65) | 5'-ATGTGGAGATCATTGAGCAGC-3' | 5'-CCTGGTCCTGTGTAGCCATT-3' | 223468680c1 |
| TLR3 | 5'-TTGCCTTGTATCTACTTTTGGGG-3' | 5'-TCAACACTGTTATGTTTGTGGGT-3' | 19718735c1 |
| TLR4 | 5'-CCTCGGCGGCAACTTCATAA-3' | 5'-AGAGCGGATCTGGTTGTACTG-3' | 116875827c1 |
| EV71 | 5'-GCAGCCCAAAAGAACTTCAC-3' | 5'-ATTTCAGCAGCTTGGAGTGC-3' | - |
| GAPDH | 5'-GGAGCGAGATCCCTCCAAAAT-3' | 5'-GGCTGTTGTCATACTTCTCATGG-3' | 378404907c1 |
| STAT1 | 5'- CGGCTGAATTTCGGCACCT-3' | 5'- CAGTAACGATGAGAGGACCCT-3' | 189458859c3 |

**Appendix 2**. The TC_50_, EC_50_ and TI values for each purification site of BBS.

| Groups | TC_50_(g/mL) | EC_50_(g/mL) | TI |
| --- | --- | --- | --- |
| S1 | 2^-0.695^ | * | * |
| S2 | 2^-1.765^ | * | * |
| S3 | 2^-1.935^ | * | * |
| S4 | 2^-1.815^ | * | * |
| S5 | 2^-4.607^ | * | * |
| S6 | 2^-3.659^ | * | * |
| S7 | 2^-2.588^ | * | * |
| S8 | 2^-1.832^ | * | * |
| S9 | 2^-5.407^ | * | * |
| S10 | 2^-0.327^ | 2^-4.643^ | 19.922 |
| S11 | 2^-0.580^ | 2^-6.000^ | 42.817 |
| S12 | 2^-0.186^ | 2^-6.556^ | 82.701 |
| P1 | 2^-6.127^ | * | * |
| P2 | 2^-3.650^ | * | * |
| P3 | 2^-5.184^ | * | * |
| P4 | 2^-3.453^ | * | * |
| P5 | 2^-3.633^ | * | * |
| P6 | 2^-5.626^ | * | * |
| Ribavirin | 2^-0.826^ | 2^-6.958^ | 70.132 |

**Appendix 3.** Validation information for the NF-*κ*B signaling pathway

**Part 1**. Standardized curve for NF-*κ*B (p65). Y: concentration of the standard (ng/L); X: the OD value of the standard.

**Part 2.** Effect of drugs on NF-*κ*B (p65) in serum of EV71-infected mice (x±s, N=5)

| Groups | A | B | C | D | E | F |
| --- | --- | --- | --- | --- | --- | --- |
| Concentration （ng/L） | 29.58±3.71 | 45.43±2.02 | 33.05±1.57^*^ | 34.12±3.06^*^ | 31.37±2.14^*^ | 37.73±1.60^*^ |

The concentration of NF-*κ*B (p65) (the results are presented as mean SD, N=5; *: P﹤0.05 compared with EV71 model group).

A: blank control group; B: EV71 model group; C: positive control group; D: high-dose group (4 g/kg/d); E: middle-dose group (2 g/kg/d); F: low-dose group (1 g/kg/d).

**Appendix 4**. Statistical chart of body weight changes in mice and the lung histopathologic sections (STAT1 mRNA groups).


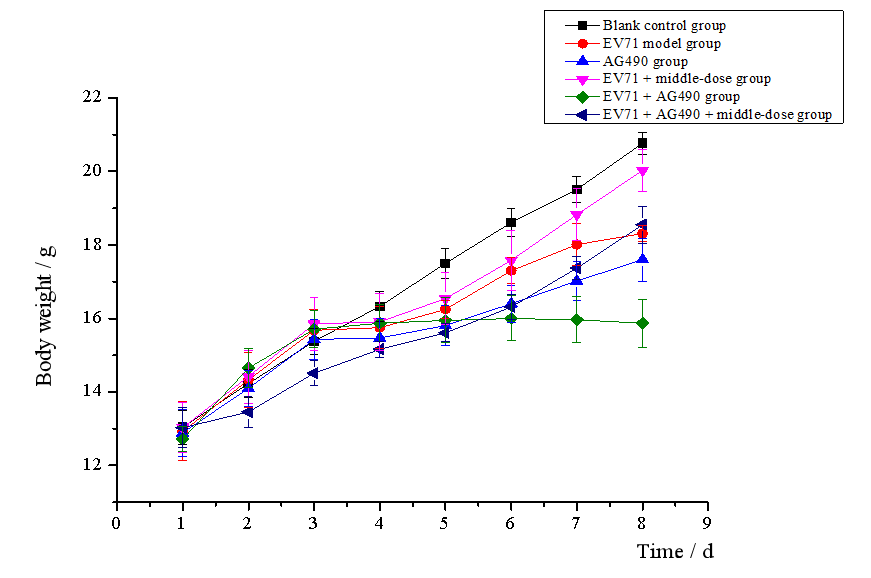


**Part 1.** The trend of body weight changes of mice (STAT1 mRNA groups).

| 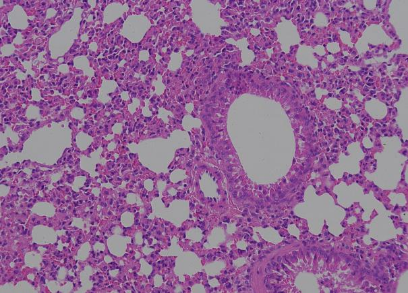 | 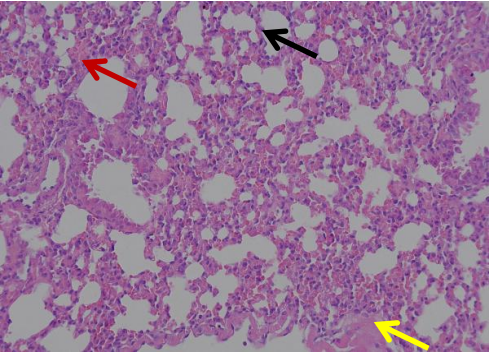 |
| --- | --- |
| **Blank control group** | **EV71 model group** |
| 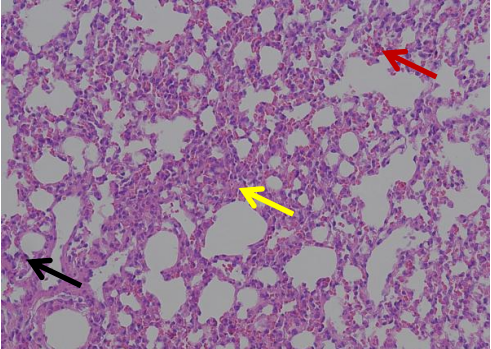 | 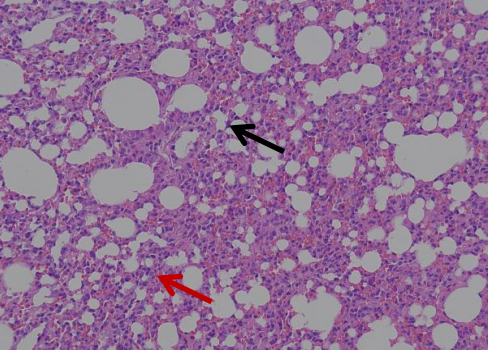 |
| **AG490 group** | **EV71 + middle-dose group** |
| 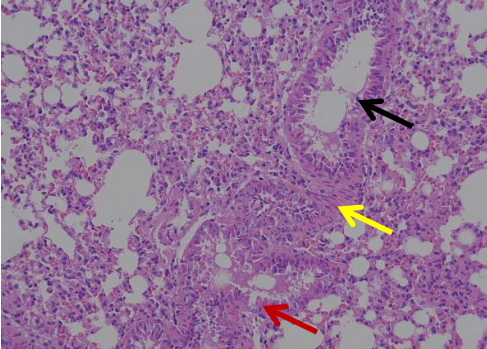 | 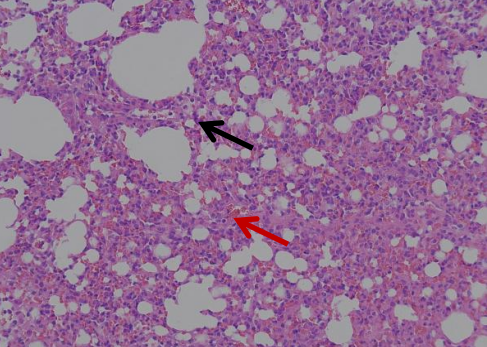 |
| **EV71 + AG490 group** | **EV71 + AG490 + middle-dose group** |

**Part 2**. Histological observation of lung tissue (200×) (STAT1 mRNA groups; black arrows: monocytes and granulocytes were infiltrated, red arrows: capillary blood clotting, yellow arrows: bronchi filled with eosinophilic secretions).

**Appendix 5**. UPLC-MS and ^1^H-NMR results of C5.

| 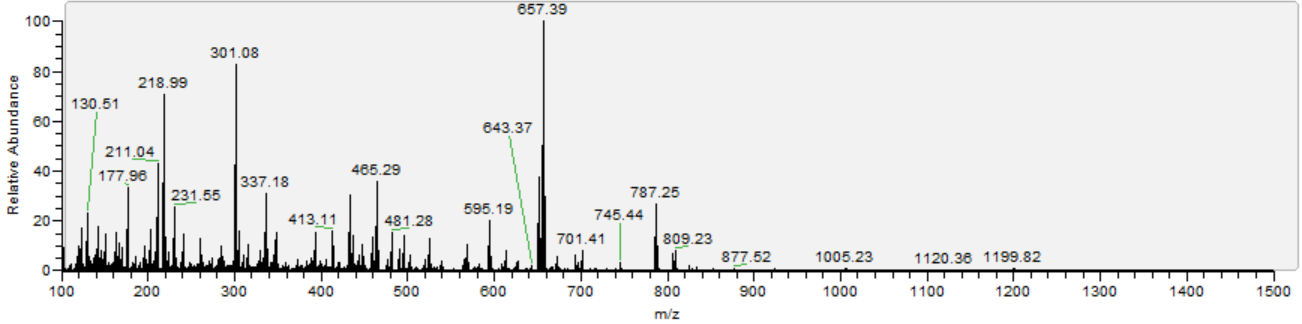  **ESI-MS [M-H]^+^** |
| --- |
| 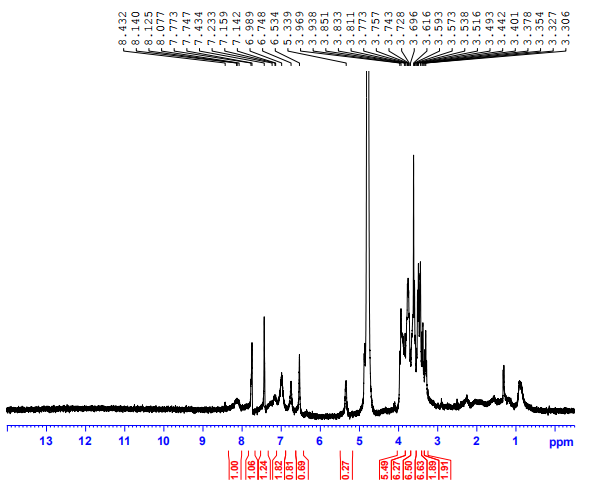  **^1^H-NMR** |

**Appendix 6.** Chromatograms of C3 and C4 and their spectral resolution.

| 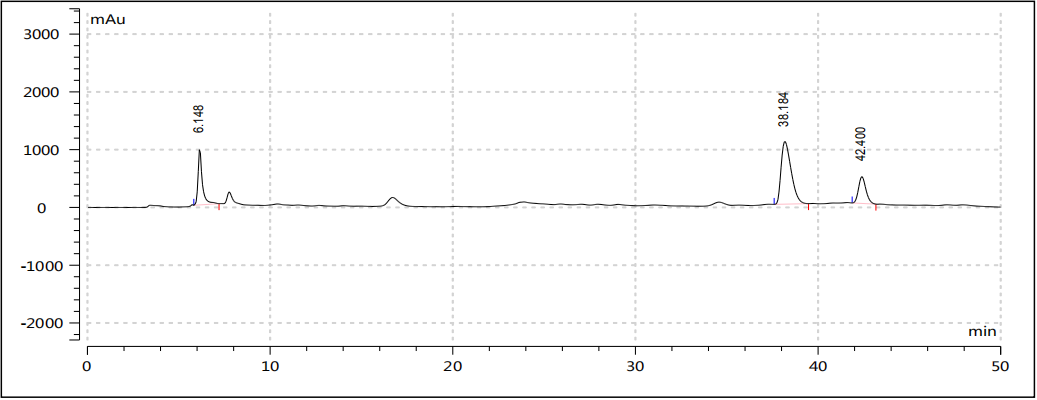  **S11** |
| --- |
| 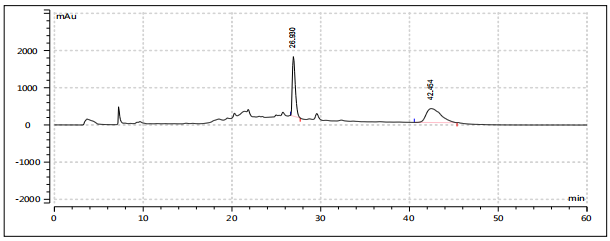  **S12** |

**Part 1**. The PLC diagrams of S11 and S12.

| 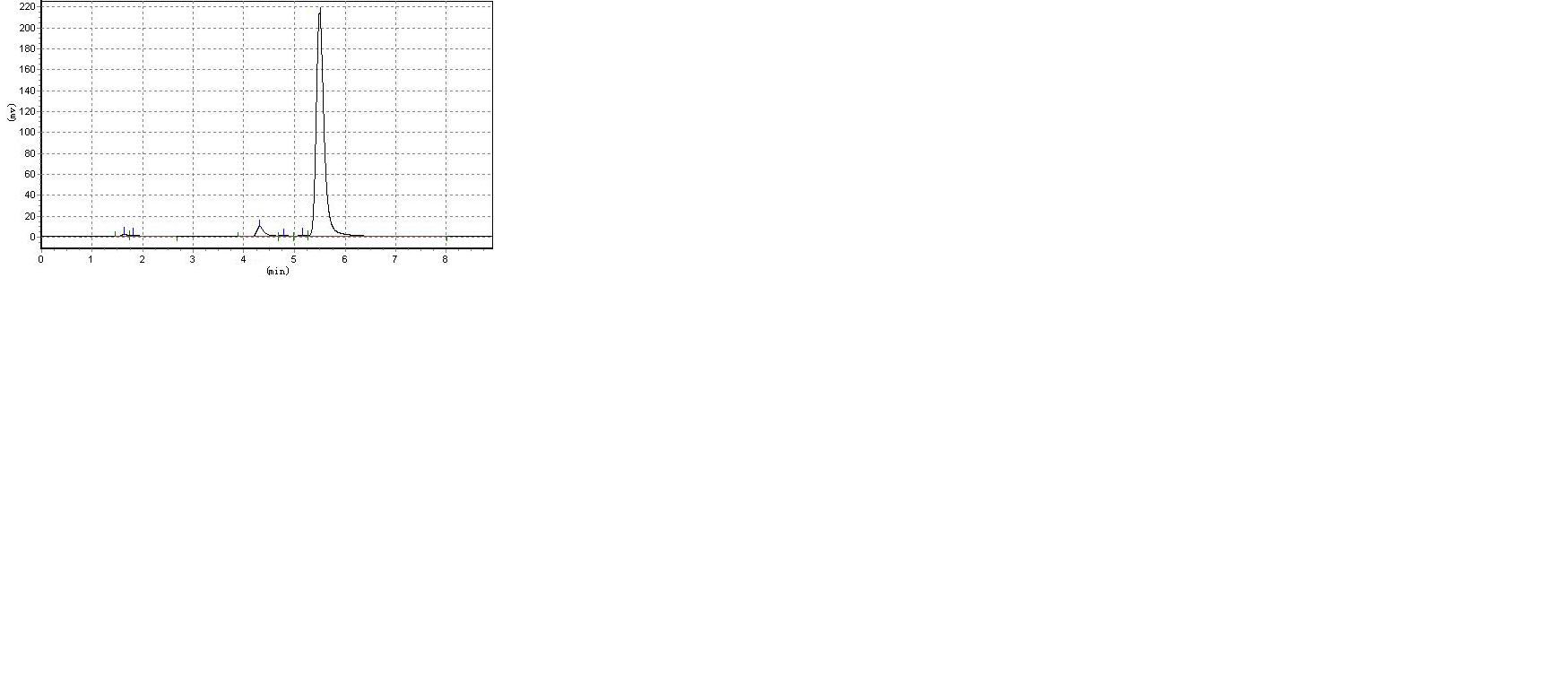  **C1** |
| --- |
| 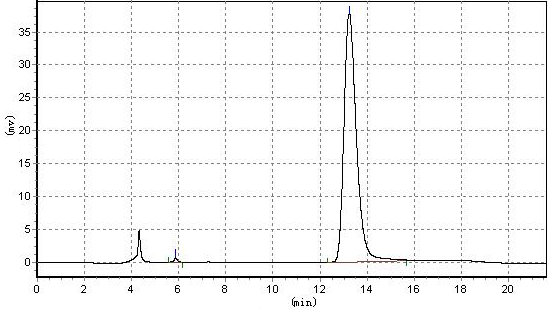  **C2** |
| 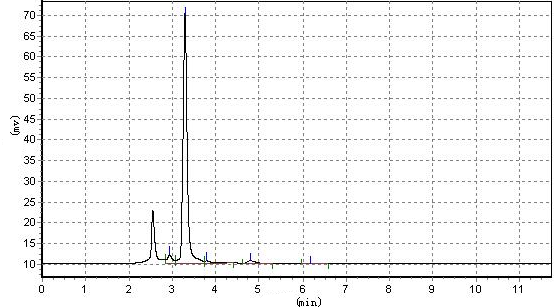  **C3** |
| 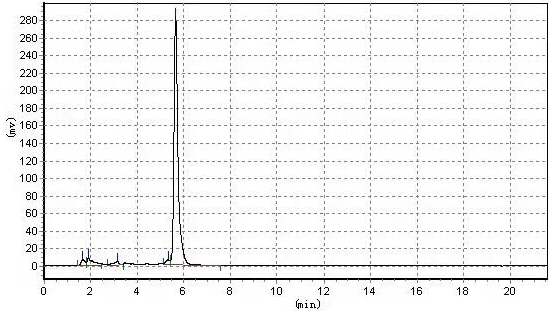  **C4** |
| 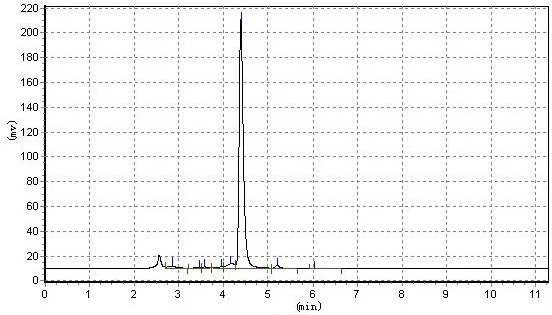  **C5** |

**Part 2**. HPLC chromatograms of each effluent monomer.

HPLC:

S11 and S12 were scanned at full wavelength under a UV spectrophotometer and their absorption was measured at 260 nm. The gradient elution was performed on the PLC on the Eclipse XDB-C_18_ column (4.6×250 mm, 5 μm) with the mobile phase of 0.1% phosphoric acid (A): acetonitrile (B) at 260 nm and the flow rate of 1.0 mL/min. The purity of each monomer (C1-C5) was determined using HPLC on the ODS-2 column (10×250 mm, 10 μm) with the mobile phase of ultrapure water (A): acetonitrile (B) = 20:80 and flow rate of 1.0 mL/min

| 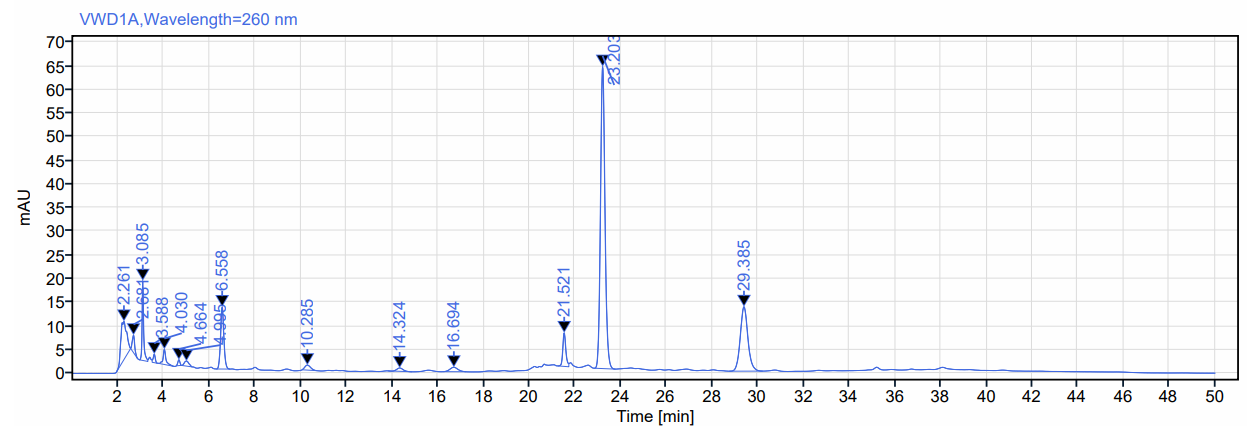  **S11** |
| --- |
| 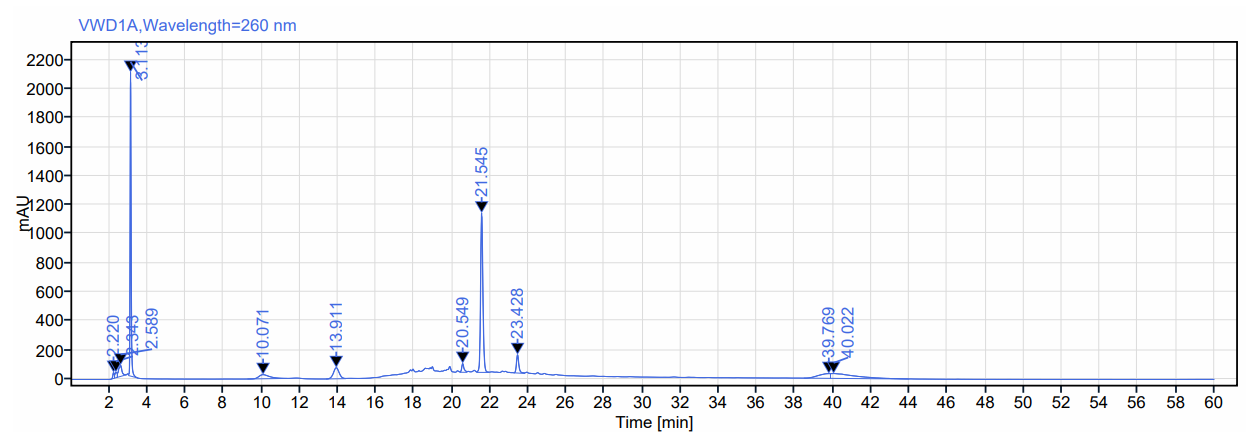  **S12** |

**Part 3.** The HPLC diagrams of S11 and S12.

The results of monomers C3 and C4’s chemical structure analysis are as follows:

ESI-MS *m*/*z*: 595 [M+H]^+^ , 593 [M-H]^−^;

^1^ H-NMR (400 MHz, DMSO-*d*6) *δ*: 13.53 (1H, s, 5-OH), 10.42 (1H, brs, 4′-OH),

7.96 (2H, d, *J* = 8.8 Hz, H-2′, 6′), 6.94 (2H, d, *J* = 8.8 Hz, H-3′, 5′), 6.91 (1H, s, H-8), 6.88 (1H, s, H-3), 4.73(1H, d, *J* = 10.1 Hz, H-1″), 4.98 (1H, d, *J* = 7.0 Hz, H-1′′′)；

^13^C-NMR (100 MHz, DMSO-*d*6) *δ*: 164.2 (C-2), 103.2 (C-3), 182.4 (C-4), 159.3(C-5), 110.6 (C-6), 162.5 (C-7), 93.7 (C-8), 156.4 (C-9), 104.9 (C-10), 120.9 (C-1′), 128.6 (C-2′, 6′), 116.0 (C-3′, 5′), 161.4 (C-4′), 101.2 (C-1′′′), 81.5 (C-5″), 78.9(C-5′′′), 77.2 (C-3″), 75.8 (C-3′′′), 73.8 (C-1″), 72.7 (C-2′′′), 70.9 (C-2″), 69.6 (C-4′′′), 69.5 (C-4″), 60.7 (C-6′′′), 60.6(C-6″).

| 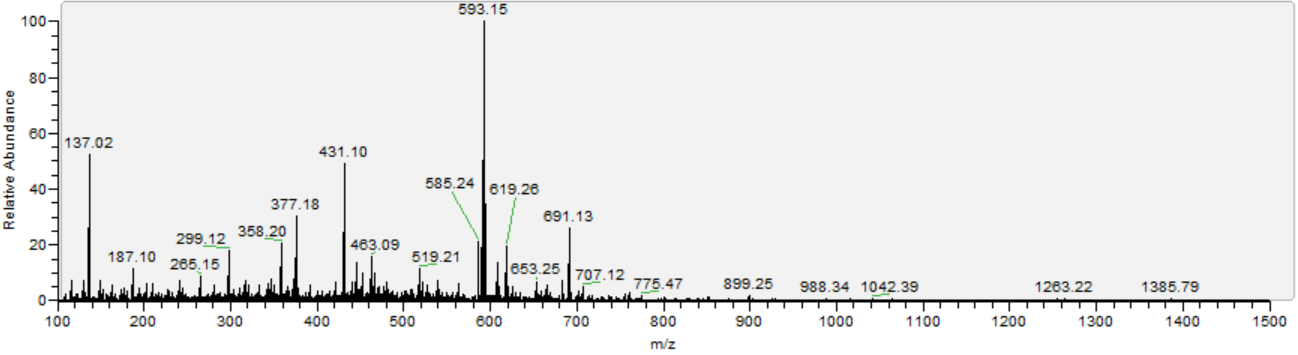  **ESI-MS [M-H]^-^** |
| --- |
| 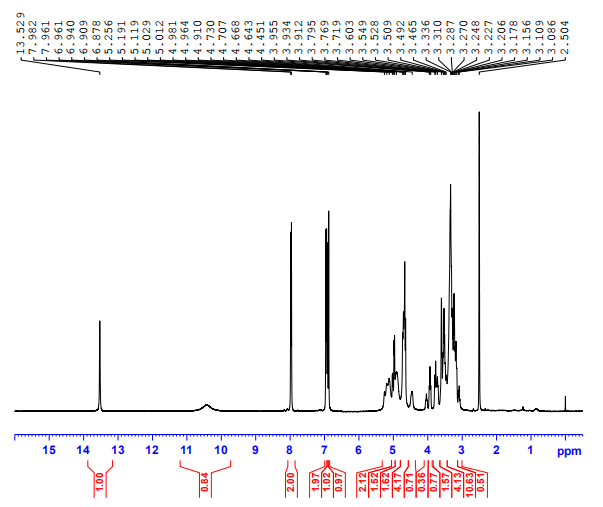  **^1^H-NMR** |
| 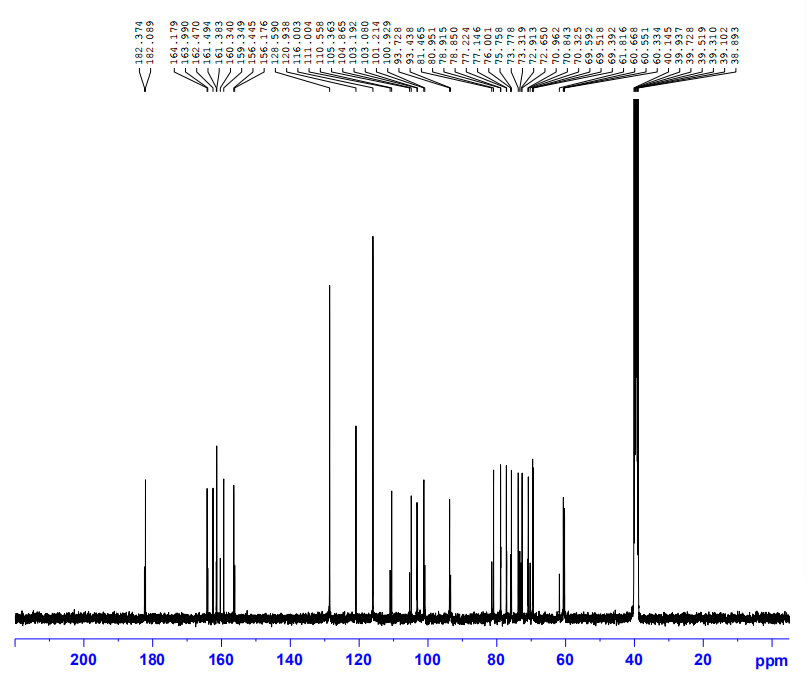  **^13^C-NMR** |

**Part 4**. UPLC-MS and NMR results of C3 and C4.

**The derivation process of the structural formula is as follows：**

**Part A:**

It indicates that the structure contains phenolic hydroxyl.

ferric trichloride reaction (+)

Target compound belongs to flavonoids.

hydrochloric acid-magnesium powder reaction (+)

Molish reaction (+)

The target compound contains glycosidic groups.

This result indicates that the compound does not contain ortho diphenol hydroxyl groups.

SrCl_2_ reaction (-)

The structure contains 8-H.

Gibb’s reaction (+)

**Part B:**

ESI-MS m/z: 595 [M+H]^+^ , 593 [M-H]^−^，these two peaks demonstrate that the mass fraction of the structure is approximately 595. Combining the data of ^1^H-NMR and ^13^C-NMR, it is inferred that its molecular formula is C_27_H_30_O_15_.

Given by ^1^H-NMR δ 6.88 (1H, s) is the characteristic signal of flavonoid compound H-3; δ 13.53 (1H, s) is a 5-OH characteristic signal associated with carbonyl groups in the A-ring; δ 10.42 (1H, brs), δ 7.96 (2H, d, J=8.8 Hz), δ 6.94 (2H, d, J=8.8 Hz) show that the B ring is a 4'-OH substituted flavonoid compound; δ 6.91 (1H, s) is the characteristic signal of H-8; δ 4.73 (1H, d, J=10.1 Hz) is a characteristic signal of glycoside terminal hydrogen bound to 7-OH, and δ 4.98 (1H, d, J=7.0 Hz) is a characteristic signal of glycosidic the first end hydrogen bound to 6-H, indicating that the A-ring in the parent nucleus of this compound is a 5,7-dihydroxysubstituted flavonoid compound, and the H in 6-H and 7-OH is replaced by glycosidic groups.

In the ^13^C-NMR spectrum δ 182.4 is the characteristic signal of the 4-position carbonyl group in flavonoids; δ 164.2 and δ 103.2 is the characteristic signal of C-2 and C-3; δ 110.6 and δ 93.7 is the characteristic signal of 5,7-dioxyflavones C-6 and C-8, respectively, and δ 159.3 and δ 162.5 is the characteristic signal of 5,7-dioxyflavones C-5 and C-7, respectively. δ 101.2 and δ 73.8 is the characteristic signal of the first end carbon in the glycosidic groups directly connected to 6-H and 7-OH in the A-ring, respectively. So the structure of the compound can be introduced as shown below:

**Appendix 7.** The TC_50_, EC_50_ and TI values for different concentration of saponarin, S12 and Ribavirin.

| Groups | TC_50_(g/mL) | EC_50_(g/mL) | TI |
| --- | --- | --- | --- |
| 1 mg/mL | 2^-0.261^ | 2^-4.997^ | 26.649 |
| 2 mg/mL | 2^-1.163^ | 2^-6.541^ | 41.585 |
| 5 mg/mL | 2^-2.245^ | 2^-8.971^ | 105.859 |
| 10 mg/mL | 2^-2.631^ | 2^-8.629^ | 63.911 |
| 50 mg/mL | 2^-2.944^ | 2^-8.531^ | 48.068 |
| S12 | 2^-0.182^ | 2^-6.512^ | 80.449 |
| Ribavirin | 2^-0.797^ | 2^-6.955^ | 71.407 |
